# Long Non-Coding RNAs as Functional Codes for Oral Cancer: Translational Potential, Progress and Promises

**DOI:** 10.3390/ijms22094903

**Published:** 2021-05-05

**Authors:** Cing-Syuan Lei, Hsing-Jien Kung, Jing-Wen Shih

**Affiliations:** 1 Ph.D. Program for Cancer Molecular Biology and Drug Discovery, College of Medical Science and Technology, Taipei Medical University and Academia Sinica, Taipei 11031, Taiwan; m507102013@tmu.edu.tw (C.-S.L.); hkung@tmu.edu.tw (H.-J.K.); 2 Graduate Institute of Cancer Biology and Drug Discovery, College of Medical Science and Technology, Taipei Medical University, Taipei 11031, Taiwan; 3 Institute of Molecular and Genomic Medicine, National Health Research Institutes, Zhunan, Miaoli County 35053, Taiwan; 4 Comprehensive Cancer Center, Department of Biochemistry and Molecular Medicine, University of California at Davis, Sacramento, CA 95817, USA; 5 TMU Research Center of Cancer Translational Medicine, Taipei Medical University, Taipei 11031, Taiwan; 6 Ph.D. Program for Translational Medicine, College of Medical Science and Technology, Taipei Medical University, Taipei 11031, Taiwan

**Keywords:** oral cancer, long non-coding RNA, tumorigenesis, cancer progression, metastasis

## Abstract

Oral cancer is one of the leading malignant tumors worldwide. Despite the advent of multidisciplinary approaches, the overall prognosis of patients with oral cancer is poor, mainly due to late diagnosis. There is an urgent need to develop valid biomarkers for early detection and effective therapies. Long non-coding RNAs (lncRNAs) are recognized as key elements of gene regulation, with pivotal roles in various physiological and pathological processes, including cancer. Over the past few years, an exponentially growing number of lncRNAs have been identified and linked to tumorigenesis and prognosis outcomes in oral cancer, illustrating their emerging roles in oral cancer progression and the associated signaling pathways. Herein, we aim to summarize the most recent advances made concerning oral cancer-associated lncRNA, and their expression, involvement, and potential clinical impact, reported to date, with a specific focus on the lncRNA-mediated molecular regulation in oncogenic signaling cascades and oral malignant progression, while exploring their potential, and challenges, for clinical applications as biomarkers or therapeutic targets for oral cancer.

## 1. Introduction

Oral cancer, one of the most common malignancies worldwide and a leading cause of mortality in certain regions, is currently a major public health issue. According to updated statistics, there was an estimated incidence of 377,000 oral cancer cases and 177,000 deaths worldwide in 2020, with a particularly high frequency in South Central Asia and Melanesia [1]. As the most common cancer developing in the head and neck region [2], oral cancer is comprised of malignancies occurring in the lips, front two-thirds of the tongue, alveolar ridge and gums, floor of the mouth, hard palate, retromolar trigone, and buccal mucosa [3,4]. Over 90% of oral malignant tumors originate from the squamous cells [5,6,7] and are collectively known as oral squamous cell carcinoma (OSCC), which can be further divided into three different subsites: buccal mucosal SCC (BMSCC), tongue SCC (TSCC), and lip SCC (LSCC). Several risk factors account for the development of oral cancer, including continuous consumption of tobacco, alcohol, or betel nut, and infection with human papilloma virus (HPV) [8]. Currently, surgery, radiation therapy, and chemotherapy are the standard primary treatments for oral cancer. Despite the advent of multidisciplinary approaches, the prognosis of advanced stage oral cancer patients is still poor, and the overall survival rate within five years remains at around 30–50% [9], mainly due to the high tendency for local recurrence, treatment resistance, and thereafter regional lymph node metastasis. At present, visual screening combined with tissue biopsy is the most common screening approach for oral cancer, but this may provide insufficient information, which results in under-diagnosis and improper care [10]. In addition, the invasive nature, high cost, site-specificity, and limited technique sensitivity make biopsy unsuitable for follow-up purposes. Meanwhile, the heterogenicity of oral cancer at the molecular level hampers the characterization of specific therapeutic targets, resulting in challenges for treatment development. Thus, an in-depth understanding about the molecular mechanisms of oral carcinogenesis and progression is an urgent priority for developing valid biomarkers for early detection and patient stratification, as well as effective therapies against oral cancer [11].

In the human genome, it has now been recognized that protein-coding transcripts represent only a tiny fraction of the transcriptional output (less than 3%), whereas the majority of transcripts encode a variety of non-coding RNAs. Among the non-coding transcripts, long non-coding RNAs (lncRNAs) are a large family of heterogeneous regulatory RNA molecules longer than 200 nucleotides and without evident protein coding potential [12,13]. According to the most updated human genome annotation (GRch38, GENCODE Release 37; www.gencodegenes.org; accessed on 15 February 2021), 48,741 transcripts originating from 17,948 loci were identified as lncRNAs. LncRNAs can be further sub-divided by their biogenesis loci, including lincRNAs (long intergenic RNAs), long intronic RNAs, eRNAs (enhancer RNAs), asRNAs (antisense RNAs), promoter RNAs, and bidirectional RNAs [14,15]. Similarly to mRNAs, most lncRNA species are transcribed by RNA polymerase II and then capped at 5′ ends and polyadenylated at 3′ ends, as well as spliced and processed. While an increasing number of lncRNAs have been identified in recent years, a steadily growing list of lncRNAs have been characterized as biochemically versatile regulators in different stages of gene expression. At epigenetic, transcriptional, and post-transcriptional levels, through interaction with other bio-macromolecules, such as chromatin DNA, RNA, and proteins, a series of lncRNAs have been shown to be capable of regulating chromatin remodeling, as well as RNA splicing, stability, modification, and translation, by acting as decoy, signals, guides, scaffolds, and sponges [13,16]. With such critical multi-functional roles in gene regulation, it is not surprising that an expanding number of dysregulated lncRNAs have been associated with cancer and demonstrated to be the driving force of malignant transformation or suppression.

**Figure 1:**
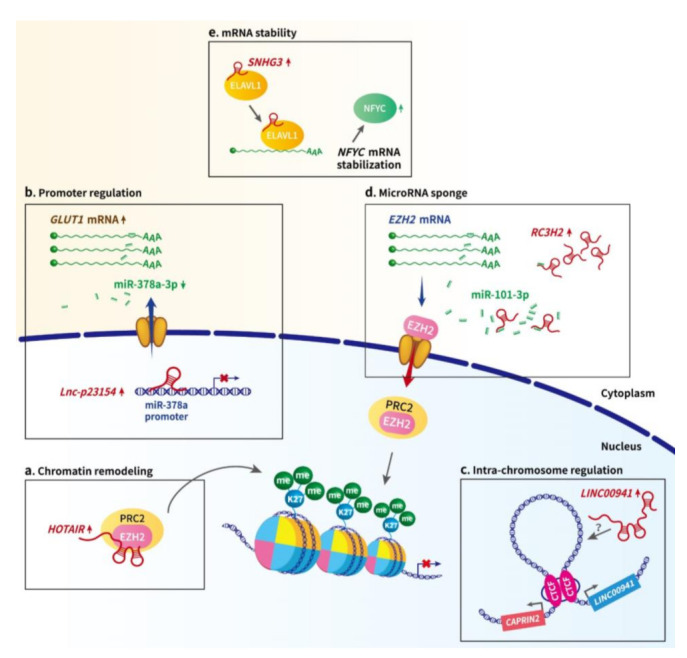
**The potential actionmechanisms of lncRNAs in oral cancer.** (**a**) **Chromatin remodeling.** LncRNA *HOTAIR* has been recognized as a scaffold interacting with chromatin modifying complexes PRC2 and LSD1 to epigenetically regulate gene expression. In OSCC, *HOTAIR* knockdown decreased the enrichment of EZH2 (the component of PRC2 complex) and H3K27me3 deposition within the E-cadherin promoter, suggesting *HOTAIR* could modify chromatin accessibility through recruiting chromatin modifying complex at the transcribed genomic locus. (**b**) **Promoter regulation.** Nuclear *lncRNA lnc-p23154* has been demonstrated to suppress miR-378a-3p transcription by interacting with its promoter region, thereby upregulating the expression of the miR-378a-3p targeted gene, *GLUT1*, and promoting Glut1-mediated OSCC metastasis. (**c**) **Intra-chromosomal interactions.** Through dimerization, transcription factor CTCF could mediate chromatin looping between its binding sites and thereby modulate transcription. LncRNA *LINC00941* could activate the expression of its nearby gene *CAPRIN2* through CTCF-mediated DNA looping of the specific region between the two genes. (**d**) **miRNA sponge/ceRNA.** LncRNA *RC3H2* could function as a miRNA sponge by physically binding miR-101-3p, whose target is *EZH2* mRNA. The upregulated EZH2 subsequently suppresses the expression of the downstream gene *CDKN2A*, facilitating the malignant behavior of OSCC cells. (**e**) **mRNA stability.** *LncRNA-SNHG3* is able to increase *NFYC* mRNA stability through interacting with RNA-binding protein ELAVL1, also known as HuR, thereby increasing NFYC protein levels.

**Figure 2:**
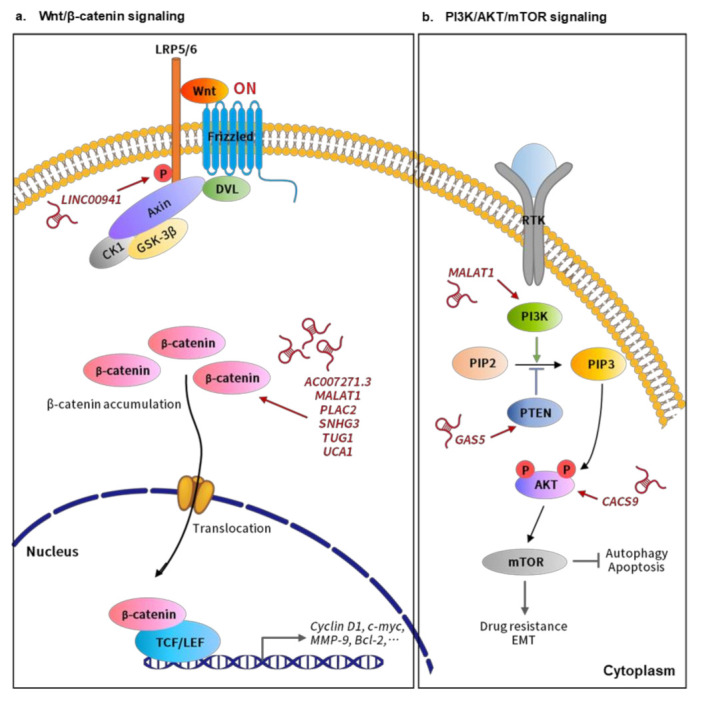
**LncRNAs involved in the relevant signaling pathways implicated in oral cancer progression.** (**a**) **Wnt/β-catenin signaling.** LncRNAs, such as AC007271.3, MALAT1, PLAC2, SNHG3, TUG1, and UCA1, could activate Wnt/β-catenin signaling mainly through inducing β-catenin accumulation. The increased β-catenin would further translocate into the nucleus and bind to LEF/TCF transcription factors to activate the downstream effectors, promoting the malignant behavior of oral cancer cells. In addition, lncRNA LINC00941 could induce the expression of Caprin-2, which further promotes the phosphorylation of the Wnt co-receptor LRP6, and thereby activating Wnt/β-catenin signaling. (**b**) **PI3K/AKT/mTOR** **signaling.** Several lncRNAs are able to target the different components of the PI3K/AKT/mTOR pathway to affect oral cancer progression. Overexpression of lncRNA MALAT1 could increase the phosphorylation of PI3K to induce PI3K/AKT/mTOR signaling, thereby promoting the EMT and cisplatin resistance of OSCC. Another lncRNA GAS5 could suppress the malignant behavior of OSCC by serving as ceRNA to sequester miR-21 and thereby reverting the miR-21-mediated repression of PTEN, a negative regulator of PI3K signaling. In addition, lncRNA CASC9 could enhance cell proliferation by inhibiting autophage-mediated cell apoptosis via inducing AKT phosphorylation and the subsequent activation of the AKT/mTOR pathway in OSCC.

Over the last few years, the advent of high-throughput sequencing techniques has enabled identification of a continuously rising amount of dysregulated lncRNAs associated with oral cancer [17,18]. Among these transcripts, only a few have been thoroughly investigated for their mechanisms of action, whereas the functional details of most remain largely uncharacterized [19,20,21,22]. Within the long-standing intense debate about the functionality of most, if not all, of these lncRNAs, a precursor–product relationship between junk RNA and functional lncRNAs has recently been suggested, in which junk transcripts may provide the raw material for the evolution of diverse lncRNAs, through a non-adaptive mechanism [23]. Overall, it is estimated that at least 87% of the transcribed regions of the human genome DNA generate junk RNAs [23]. Along these lines, most unannotated lncRNAs overexpressed in oral cancer could be simply junk transcripts produced from the accelerated transcription of tumor cells. The clinical relevance and functionality of these so-called junk lncRNAs await future investigation. Notably, the aberrant expression of numerous oral cancer-associated lncRNAs has been significantly linked to the clinicopathological features and survival outcomes of patients, revealing that functional annotation of these transcripts may eventually lead to the development of early diagnosis and new avenues for oral cancer treatment. In the present review, we aim to provide a systematically updated overview of the current knowledge about oral cancer-associated lncRNAs, summarizing their dysregulation and the potential mechanisms (Table 1 and Figure 1), and with a specific emphasis on the functional involvement of these lncRNA species in pivotal oncogenic signaling pathways (Figure 2) and the perspectives of these lncRNAs for possible clinical applications.

## 2. Mechanism of Oral Cancer-Associated lncRNAs in Tumorigenesis

Tumorigenesis is a complicated multifaceted process that involves three major stages: initiation, progression, and metastasis. Notably, in the past decade, accumulating studies have shown that a series of oral cancer-associated lncRNAs are closely connected to each step of tumorigenesis through their multiple regulatory roles in gene regulation, at various stages, such as chromatin remodeling, transcription activation, RNA interference, and RNA splicing, as well as post-translational control. Table 1 presents an updated list of these oral cancer-associated lncRNAs. In tumor tissues, most oral cancer-associated lncRNAs are significantly up-regulated, whereas *C5orf66-AS1* [24], *CASC2* [25,26,27], *ENST00000470447*.1 [28], *FALEC* [29], *LINC01315* [30], and *MORT* [31] are among the few which are down-regulated in oral cancer tissues. Although the mechanistic details might remain unclear, most of the oral cancer-associated lncRNAs have an impact on the oral tumorigenesis process. Table 1 summarizes their potential molecular targets along with their reported functions. Notably, several of them, including *ANRIL*, *CASC2*, *CCAT1*, *FGD5*-AS1, *LncHIFCAR*, *HOTAIR*, *HOTTIP*, *HOXA11*-*AS*, *MALAT1*, *MCM3AP*-*AS1*, *MEG3*, *OIP5*-*AS1*, *PVT1*, *SNHG20*, *SNHG3*, *TUG1,* and *UCA1,* might adopt more than one action mode. For the sake of conciseness, a few selected oral cancer-associated lncRNAs are enumerated below to illustrate their mechanisms of actions.

### 2.1. LncRNA-Mediated Epigenetic and Transcriptional Regulation in Oral Cancer

Chromatin remodeling is a complicated process, dynamically altering nucleosome structure by modification of the chromatin architecture to modulate access of genomic DNA to the transcription machinery during gene expression control of the eukaryotic genome. Among various nucleosome remodeling modifications, histone modifications have been functionally linked to epigenetic gene regulation. To date, two lncRNAs, *HOTAIR* (HOX transcript antisense RNA) and *FALEC* (focally amplified long non-coding RNA in epithelial cancer), have been proposed to impact the histone modification and transcriptional state in OSCC [29,32]. *HOTAIR* is an oncogenic molecule in a variety of cancers, and functions as a molecular scaffold connecting the histone modification complexes PRC2 (polycomb repressive complex 2) and LSD1 (lysine specific demethylase 1), thereby regulating gene expression via modulation of histone modifications [33,34]. In OSCC cells, Wu et al. found that *HOTAIR* could repress E-cadherin expression, whereas *HOTAIR* knockdown would impair the binding of EZH2 (the functional enzymatic component of PRC2) and H3K27me3 within the E-cadherin promoter, suggesting *HOTAIR* suppresses E-cadherin expression partly through associating with EZH2 [32] (Figure 1a). Subsequent studies further demonstrated that the upregulation of *HOTAIR* is closely associated with the progression and poor prognosis of OSCC patients [32,35,36,37]. As another example, in TSCC, the tumor suppressive lncRNA *FALEC* could recruit EZH2 at the promoter regions of the oncogene *ECM1* (extracellular matrix protein 1), epigenetically repressing ECM1 expression, and thereby repressing malignant behaviors [29].

Beyond histone modifications, a possible lncRNA-mediated crosstalk between chromatin organizer and transcription machinery has also been proposed. The DNA-binding protein CTCF (CCCTC-binding factor) is a key player in chromatin organization. By homodimerization of the CTCF proteins and working together with cohesin, CTCF is thought to regulate DNA looping and mediate the 3D structure of chromatin. Recent studies have shown that lncRNAs can regulate gene expression by altering the chromatin architecture or driving the eviction of architectural proteins, including CTCF [38]. In OSCC, Ai et al. demonstrated that oncogenic *LINC00941* is highly upregulated due to EP300-driven transcriptional activation, through enhancing H3K27ac deposition within its promoter. Most notably, upregulated *LINC00941* could in turn activate the expression of its nearby gene *CAPRIN2*, whereas chromatin organizer CTCF was required for this *LINC00941*-induced CAPRIN2 overexpression. As CTCF has been shown to mediate the looping of the chromatin fragment between *LINC00941* and *CAPRIN2* genes through its binding sites being enriched within this region, these results suggested that *LINC00941* might induce CAPRIN2 expression through CTCF-mediated DNA looping [39] (Figure 1b).

In addition, lncRNAs have been shown to transcriptionally modulate the tumorigenesis of OSCC through their interactions with DNA or protein molecules. Wang et al. demonstrated that the lncRNA *lnc-p23154*, which is mainly localized in the nucleus, suppresses miR-378a-3p transcription via binding to the miR-378a-3p promoter (Figure 1c). As miR-378a-3p could repress Glut1 expression by targeting its 3ʹUTR, the *lnc-p23154*-mediated miR-378a-3p downregulation consequently leads to increased Glut1 expression and glycolysis, which in turn accelerates OSCC metastasis [40]. Meanwhile, Zhu et al. found that, in OSCC cells, lncRNA *HAS2-AS1* (hyaluronan synthase 2 antisense 1) could promote hypoxia-induced cancer progression via inducing the expression of *HAS2* (hyaluronan synthase 2) [41]. Since an RNA–DNA interaction between the *HAS2-AS1* and *HAS2* genes has been reported previously [42], these findings suggested the binding of *HAS2-AS1* to *HAS2* gene is necessary for HAS2 upregulation. However, whether or not some other chromatin modifiers could cooperate with this RNA–DNA structure to elicit epigenetic modifications remains to be investigated.

Moreover, in our previous report, hypoxia-induced lncRNA *LncHIFCAR* (long noncoding HIF-1α co-activating RNA; also named as *MIR31HG*) was found to directly interact with HIF-1α, which in turn facilitated the formation and recruitment of HIF-1 complex to the promotor region of metastasis-driving genes, and thereby promoting their transcription, as well as OSCC progression [43]. Several lines of evidence in our current study revealed the participation of some other histone modifiers in *LncHIFCAR*-mediated HIF-1 co-activation (unpublished data), which warrants further investigation. Moreover, in LSCC, other than HIF-1α, *MIR31HG* was demonstrated to target p21 to promote the tumorigenic process through an uncharacterized mechanism [44]. Consistent with the above findings, these studies collectively noted that *MIR31HG*/*LncHIFCAR* could serve as a poor prognostic factor and putative therapeutic target in HNSCC [43,44,45].

### 2.2. LncRNA-Mediated Post-Transcriptional Regulation in Oral Cancer

Recent studies have validated that a set of lncRNAs can collaborate with mRNAs or pre-mRNAs to form “lncRNA–mRNA pairs” and consequently fine-tune the mRNA splicing or stability [46,47]. In OSCC tissues, Guo et al. identified a highly upregulated lncRNA *CEBPA-AS1* (also known as *LOC80054*) as a potential oncogene and a prognostic biomarker, the expression of which correlates with poor differentiation, lymph node metastasis, and high clinical stage [48]. *CEBPA-AS1* knockdown inhibited the tumorigenesis of OSCC by reducing the expression of its nearby gene, *CEBPA*. Mechanistically, *CEBPA-AS1,* which is predominantly localized in the cytoplasm and the perinuclear region, was found to interact with *CEBPA* mRNA directly, suggesting that *CEBPA-AS1* may induce *CEBPA* expression in cis through this unique “lncRNA (*CEBPA-AS1*)-mRNA (CEBPA)” pairing [48]. However, the mechanistic details still need to be elucidated.

Furthermore, the hypothesis of competing endogenous RNA (ceRNA) proposed by Salmena et al. in 2011 [49] has generated substantial interest in uncovering lncRNA function through the ceRNA mechanism. Indeed, some abundant lncRNAs harboring miRNA-complementary sites are able to modulate gene expression as ceRNAs or “miRNA sponges”, thereby sequestering miRNAs and neutralizing the miRNA-mediated negative regulatory effects on their target RNAs [13]. However, the stoichiometric relationship between a miRNA and its target sites presented on the potential competitive endogenous lncRNA is critical for achieving a measurable effect on target–mRNA expression [50,51,52]. Recently, a large number of lncRNAs have been reported to function as miRNA sponges, reducing miRNA availability to target mRNAs in OSCC. For example, by multi-transcriptome analysis, bioinformatics analysis, and qRT-PCR, Wu et al. validated a co-expression network among lncRNA *RC3H2*, EZH2, and miR-101-3p. Notably, the cytoplasmic abundant lncRNA, *RC3H2,* could physically interact with miR-101-3p, while suppression of miR-101-3p could attenuate the lncRNA *RC3H2* knockdown-induced inhibitory effects on OSCC cells by targeting EZH2, revealing that lncRNA *RC3H2* could act as ceRNA to up-regulate EZH2 expression by sponging miR-101-3p (Figure 1d), which subsequently affected the level of H3K27me3 deposition and the expression of downstream genes associated with cancer progression [53,54]. Moreover, the *MEG3* [55], *ANRIL* [56], *FGD5-AS1* [57], *H19* [58], *HOTAIR* [37], *LINC01315* [30], *MALAT1* [59], *PDIA3P* [60], *RBM5-AS1* [61], *SNHG20* [33], *TUG1* [62], and *TTN-AS1* [63] lncRNAs were reported to have the ability to physically interact with miRNAs and thereby influence the development of OSCC. Considering the requirements for appropriate experimental manipulations [52], such as the well-controlled overexpression of miRNAs within physiological ranges and additional evidence supported by miRNA suppression experiments to avoid the potential saturation of RISC complexes, the claims of ceRNA interactions may require more critical evaluation.

Notably, through interaction with a variety of RNA binding proteins (RBPs), lncRNAs are known to participate in the regulation of RNA splicing and stability [64]. For instance, the RNA binding protein ELAVL1 (embryonic lethal abnormal vision-like protein 1), also known as HuR (Hu antigen R), is crucial for the nuclear import and stabilization of numerous RNA transcripts [65]. In OSCC, the oncogenic lncRNA *SNHG3* (small nucleolar RNA host gene 3) was reported to be localized in the cytoplasm and capable of recruiting ELAVL1, and thereby stabilizing *NFYC* (transcription factor Y subunit gamma) mRNA to upregulate the expression of NFYC, which in turn promotes cell proliferation and migration [66] (Figure 1e).

Collectively, through interplay with different types of bio-molecules, oral cancer-associated lncRNAs engage in a variety of critical steps in oral carcinogenesis. These findings substantiate the potential of these lncRNAs as key regulators of oral tumorigenesis. Of note, several other kinds of functional mechanism of lncRNAs have not yet been reported in oral cancer. For example, a few lncRNAs harboring small open reading frames (sORFs) could be translated into micro-peptides (usually fewer than 100 amino acids), whereas some tumor-related functional peptides are reported to regulate biological processes and influence tumorigenesis and progression steps [67,68]. In addition, another class of lncRNAs are responsible for alternative splicing during tumorigenesis, through regulating the phosphorylation status or hijacking splicing factors [47]. With the variety of novel mechanisms described above, additional mechanistic scenarios adapted by lncRNAs in oral cancer are expected to be unraveled in the near future.

## 3. LncRNAs Involved in Crucial Signaling Pathways

It is now well appreciated that the genetic alterations in signaling pathways responsible for cell proliferation, cell-cycle progression, and cell death are common hallmarks of cancer. Currently, targeting the oncogenic components in these pathways is a pertinent therapeutic strategy for a variety of tumors, especially for enhancing the chemo-sensitivity and avoiding drug resistance. In oral cancer, a diverse array of pivotal signaling pathways, including the Wnt/β-catenin signaling PI3K/AKT/mTOR pathway and others, have been identified as being frequently genetically altered [136]. Emerging evidence has demonstrated that certain lncRNAs could participate in the pathogenesis of oral cancer through these downstream pathways. Here we highlighted the roles of those lncRNAs involved in oncogenic signaling pathways during oral tumorigenesis, which may assist in the discovery of reliable lncRNA-based diagnostics, and preferably, therapeutic strategies for this disease.

### 3.1. Wnt/β-Catenin Signaling-Related lncRNAs in Oral Cancer

Wnt signaling, categorized into canonical β-catenin-dependent and non-canonical β-catenin-independent pathways, is an evolutionarily conserved cascade known to be involved in proliferation, regeneration, differentiation, stemness, and development. Recent studies have revealed the emerging prominent oncogenic role of aberrant Wnt/β-catenin signaling in OSCC tumorigenesis [137]. In the best studied canonical β-catenin-dependent pathway, Wnt ligands signal across cell membranes by binding the frizzled receptors and coreceptor LRP (low density lipoprotein receptor-related protein), resulting in the activation of the disheveled proteins (DSH) to phosphorylate LRP5/6 (lipoprotein receptor-related proteins 5/6), which in turn disassembles the β-catenin destruction complex containing GSK3β (glycogen synthase kinase-3β), APC (adenomatous polyposis coli), CK1 (casein kinase 1), and axin (Figure 2a). This leads to stabilization and accumulation of β-catenin, which subsequently translocates into the nucleus, where nuclear β-catenin forms a transcriptional complex with the TCF (T-cell factor)/LEF (lymphoid-enhancing factor) family of transcription factors, thereby activating the expression of downstream genes, including *c-Myc*, *Bcl-2,* and *Cyclin D1*, to promote tumorigenesis (Figure 2) [138].

Currently, several lines of evidence have demonstrated that a series of lncRNAs participate in Wnt/β-catenin signaling in oral carcinogenesis. For instance, in TSCC, the aberrant upregulation of lncRNA *UCA1* (urothelial carcinoma-associated 1) was found to be associated with metastasis and TNM classification [135]. *UCA1* could activate the WNT/β-catenin signaling pathway and the expression of its downstream targets, such as Cyclin D1 and MMP-9, by promoting the translocation of β-catenin into the nucleus, although the underlying mechanism remains unclear. Similarly, lncRNAs *AC007271.3* [70], *MALAT1* [107], *PLAC2* (placenta-specific protein 2) [120], *SNHG3* (small nucleolar RNA host gene 3) [66], and *TUG1* (taurine upregulated gene 1) [131] have been reported to induce the upregulation of β-catenin and subsequent activation of the WNT/b-catenin signaling downstream genes in oral cancer (Figure 2a), though the mechanistic details remain to be further investigated.

As mentioned above, recent studies have identified a large number of lncRNAs acting as miRNA sponges to absorb miRNAs, interfering with their regulatory effects on the downstream mRNA targets. Indeed, in OSCC, lncRNA *CCAT1* (colon cancer associated transcript 1) was found to activate Wnt/β-catenin signaling via its direct interaction with miR-181a, a suppressor of Wnt inhibitory factor-1 (WIF) [139], and therefore driving cell proliferation, migration, and invasion [78]. Most remarkably, another novel lncRNA, *TIRY*, was found to be highly overexpressed in cancer-associated fibroblasts (CAFs) from OSCC tissues [130]. Nowadays, cancer-associated fibroblasts (CAFs) are considered as one of the most pivotal and abundant components of the tumor microenvironment, contributing to the tumorigenic features of oral cancer [140]. Notably, Jin et al. demonstrated that *TIRY*-overexpressing CAFs could promote the invasive phenotypes of OSCC cells through CAFs-derived exosomes secretion [130]. Subsequent analysis showed that lncRNA *TIRY* could physically bind to miR-14 and lead to insufficient miR-14 expression in exosomes derived from CAFs, whereas *WNT3A* mRNA was predicted as the target of miR-14. Collectively, these results implied that *TIRY* overexpression interferes with the production of miR-14 in CAF-derived exosomes, which in turn results in the activation of Wnt/β-catenin signaling and the enhanced invasion and metastasis of OSCC [130]. This study not only supports the involvement of lncRNAs in Wnt signaling regulation, but also reveals the potential therapeutic value of CAFs. Meanwhile, the aforementioned lncRNA *LINC00941* could provoke canonical Wnt signaling via upregulating the expression of Caprin-2 [39], a positive regulator of Wnt signaling, by promoting GSK3-depedent LRP5/6 phosphorylation (Figure 2a), and thereby promoting OSCC progression [141].

### 3.2. PI3K/AKT/mTOR Signaling-Related lncRNAs in Oral Cancer

The phosphoinositide 3-kinase (PI3K)/AKT/mammalian target of rapamycin (mTOR) pathway, a complicated intracellular signaling cascade involved in multiple cellular processes, such as survival, growth, proliferation, autophagy, apoptosis, angiogenesis, and metabolism, is aberrantly hyper-activated in many cancer types, including oral cancer [142]. Upon initiation, various extracellular growth factors and ligands interact with their respective transmembrane receptor tyrosine kinases (RTKs), leading to subsequent phosphorylation of PI3K and producing the secondary messenger, phosphatidylinositol 3,4,5-triphosphate (PIP_3_), which in turn activates its downstream effectors, including Akt and mTOR. Notably, AKT activity is a central determinant in the PI3K pathway. PI3K serves as a positive AKT regulator, whereas PTEN (phosphatase and tensin homolog), a tumor suppressor, is a negative PI3K regulator [143,144]. During the last decade, more and more lncRNAs have emerged as critical regulators of PI3K/AKT/mTOR signaling in oral tumorigenesis.

Currently, for the treatment of OSCC, cisplatin (DDP) is the first-line chemotherapy agent, whereas acquired DDP resistance greatly diminishes drug efficacy and survival benefit. In DDP-resistant OSCC, Wang et al. reported the increased expression of lncRNA *MALAT1*. Specifically, by the activation of PI3K/AKT/mTOR signaling pathway, *MALAT1* overexpression could lead to DDP resistance, whereas *MALAT1* knockdown could effectively re-sensitize OSCC cells to DDP treatment, suggesting an instrumental role of *MALAT1* in PI3K/AKT/mTOR signaling-associated DDP resistance development [108]. Furthermore, PI3K/AKT/mTOR signaling is also known as a critical regulatory pathway of autophagy, a highly conserved catabolic process involving the lysosome-mediated degradation of intracytoplasmic components and participating in a variety of cellular biological activities [145]. Autophagy may block or promote tumor survival, depending on the various tumor types and stages. Recently, in OSCC, another lncRNA *CASC9* was found to promote cancer progression through suppressing autophagy-mediated cell apoptosis via inducing AKT phosphorylation and the subsequent activation of the AKT/mTOR pathway [75] (Figure 2b). In contrast, the tumor suppressive lncRNA *GAS5* (growth-arrest-specific transcript 5) could suppress proliferation, migration, invasion, and epithelial-mesenchymal transition (EMT) in OSCC [86,87]. Mechanically, *GAS5* has been reported to act as a miR-21 sponge in ovarian and cervical cancer. In OSCC, Zeng et al. observed that *GAS5* most likely also functions in this way. By sequestering miR-21, *GAS5* rescues the expression of PTEN, a negative regulator of PI3K signaling, from miR-21-mediated repression, and thereby inhibiting the PI3K/AKT pathway [87] (Figure 2b). Taken together, accumulating reports have revealed the prominent involvement and implications of lncRNAs in regulating cellular proliferation, drug resistance, and apoptosis through targeting the PI3K/AKT/mTOR signaling cascade during oral carcinogenesis.

### 3.3. Other Oncogenic Signaling Pathways-Related Lncrnas in Oral Cancer

In oral cancer, multiple lncRNAs have also been reported to participate in other oncogenic signaling pathways. For example, TGF-β is known to be involved in numerous cellular processes, including proliferation, differentiation, apoptosis, homeostasis, epithelial-mesenchymal transition (EMT), and migration. Dysregulation of TGF-β signaling has been implicated in carcinogenesis and could cooperate with other oncogenic cascades to facilitate the development of aggressive tumors [146,147]. Notably, two lncRNAs *PAPAS* (promoter and pre-rRNA antisense) [119] and *ANRIL* (antisense non-coding RNA in the INK4 locus) [71] were recently reported to stimulate TGF-β signaling, thus promoting tumor progression, although the underlying mechanism remains uncharacterized. Moreover, a positive regulatory effect of the oncogenic, STAT3-induced lncRNA *HNF1A-AS1* (hepatocyte nuclear factor 1 homeobox A-antisense RNA 1) on Notch signaling was demonstrated to promote OSCC progression [89]. Nevertheless, another tumor suppressive lncRNA, *MEG3*, could suppress the JAK-STAT (Janus kinases-signal transducer and activator of transcription) pathway via sponging miR-548d-3p that targets SOCS (suppressor of cytokine signaling proteins), SOCS5, and SOCS6, and thereby inhibiting migration and promoting apoptosis in OSCC [112]. Of note, in OSCC, certain lncRNA–protein interactions could interfere with pivotal signal transduction in tumorigenesis and progression. It has been reported that oncogenic lncRNA *LEF1-AS1* (lymphoid enhancer-binding factor 1 antisense RNA 1) participated in the Hippo signaling pathway via interacting with large tumor suppressor kinase 1 (LATS1). Once *LEF1-AS1* was silenced, the abolition of the interaction between *LEF1-AS1* and LATS1 resulted in elevated binding of LATS1 to the other protein partner, monopolar spindle-one-binder (MOB), triggering the phosphorylation of the carcinogenic transcriptional coactivators YAP1 (yes-associated protein 1) and impairing its nuclear translocation, thereby inhibiting cell survival, proliferation, and migration [98].

Collectively, recent advances in molecular characterization have begun to unravel the functional involvement of lncRNAs in the critical signaling pathways in oral tumorigenesis. This knowledge will surely contribute to our improved understanding of OSCC pathogenesis and can be utilized to develop personalized combinatorial therapeutic strategies.

## 4. LncRNAs as Potential Biomarkers and Therapeutic Targets for Oral Cancer: Challenges and Potential

A biomarker, according to the current definition by the Food and Drug Administration (FDA), is any characteristic indicating normal or pathogenic processes, or any response to therapeutic interventions and exposure [148]. With the concept of precision medicine in cancer therapy, the use of disease-specific biomarkers could help to adjust treatments to individual, or subgroups of, patients. Compared with the common cancer biomarkers used in recent clinical applications, such as tumor-associated enzymes and metabolites, increasing evidence suggests that lncRNAs could be promising biomarkers in cancer diagnosis and prognosis, due to their unique RNA properties of easy detection, more tissue-specific expression, and more stable structure [149,150].

Traditionally, in clinical examination, tissue biopsy is employed as an effective and reliable diagnostic and prognostic tool. According to recent reports, an array of lncRNAs have been reported for their potential adaption to the diagnosis and prognosis of oral cancer. Compared with normal tissues, most of the oral cancer-associated lncRNAs reported so far exhibit higher expression levels in tumor tissue, while some of them are downregulated (Table 1), and are considered to be promising potential biomarkers for tissue biopsy. As more and more dysregulated lncRNAs are found to cause, or be linked to, tumor aggressiveness, further characterization of their differential expression levels in different clinical stages, combined with the existing protein-coding biomarkers could facilitate the development of novel markers of tumor aggressiveness. Furthermore, considering their tissue-specific expression patterns and involvement in pivotal oncogenic signaling pathways, lncRNAs could be utilized for specific cancer subtype diagnosis and targeted therapy [20]. Meanwhile, considering the high degree of intratumor heterogeneity in oral cancer, it would be critical to develop quantitative approaches which could detect not only the abundance, but also the heterogeneity and the spatial distribution, of clinically relevant biomarkers inside tumor samples. In particular, recent advances in in situ RNA fluorescence hybridization assays allow detecting single RNA molecules with high specificity [151,152]. In theory, these microscopical analyses could enable the visualization and accurate quantification of clinically relevant lncRNA biomarkers across entire FFPE tissue sections at a single-cell resolution, providing a robust approach for measuring the spatial heterogeneity in FFPE tissue specimens.

However, several drawbacks, such as the requirement for operation, discomfort of the patients, along with the risk and complexity of sample collection, make tissue biopsy an impractical option for the monitoring of patient health over time [153]. Instead, liquid biopsies, the minimally invasive sampling from cell-free fluids, such as blood, urine, saliva, cerebrospinal fluid (CSF), sputum, stool, and pleural effusions [154,155,156] for the subsequent analysis of “tumor circulome”, are emerging as a favorable alternative to conventional tissue biopsies [154,157]. The “tumor circulome” is defined as the subset of circulating components derived from tumor tissues that can be utilized as a reservoir of cancer biomarkers in liquid biopsies [158]. These components include tumor-derived proteins, circulating tumor cells (CTCs), extracellular vesicles (EVs), and extracellular RNAs [154,157].

Over the past few years, the growing number of extracellular lncRNAs found in the body fluids of cancer patients has encouraged more and more investigations. Using deep sequencing methods, altered expression of extracellular lncRNAs has been identified in different types of cancers, which could be of potential clinical relevance. Currently, blood, including plasma or serum, is the most widely used biological specimen in liquid biopsy biomarker development. In oral cancer, rapid expanding experimental data and potential biomarkers have been accumulated and reported. In a cohort comprising 80 OSCC patients and 70 healthy control individuals, Shao et al. demonstrated that aberrant *AC007271.3* lncRNA expression in OSCC was significantly correlated with clinical stage, especially in early-stage [69]. Of particular note, serum *AC007271.3* level could discriminate between OSCC and normal controls with sensitivities of 77.6% and specificities of 84.5%, whereas the combined detection of serum *AC007271.3* with conventional biomarkers, such as SCCA (squamous cell carcinoma antigen) and TSGF (tumor-specific growth factor), could make further differentiation of these two groups [69], uncovering a promising circulating lncRNA biomarker for OSCC, although further validation with a larger cohort is needed. Apart from *AC007271.3*, serum lncRNA *LOC284454* [159] and plasma *CACS15* [76] were also reported to be capable of serving as potential diagnostic biomarkers for OSCC.

In oral cancer, considering its direct contact with oral cancer lesions, as well as the convenience, economy, and safety of the sampling, manipulation, and preservation of saliva, saliva is becoming an attractive alternative to tissue and blood testing. Indeed, with the help of novel approaches, including metabolomics, genomics, proteomics, and bioinformatics, the saliva research field is rapidly advancing, with the aim of developing non-invasive salivary biomarkers, such as circulating tumor DNA, extracellular vesicles, miRNAs, and circulating tumor cells, into an effective modality for the early diagnostic detection, prognostic prediction, and continuous post therapy surveillance of this dread malignancy [160]. In the case of salivary lncRNA, Tang et al. investigated the levels of six well-documented cancer-associated lncRNAs in saliva collected from nine OSCC patients. In this pilot report, certain lncRNAs, like *HOTAIR* and *MALAT1,* were detectable in their settings and appeared to be potential markers for OSCC diagnosis [161]. However, due to the low abundance of lncRNAs in saliva, specific detection remains a challenge and further improvements should be made before introducing salivary lncRNAs into clinical practice. The conventional analytical Q-PCR methods for salivary analysis, involving many steps, may require further optimization, such as the standardization of sample preparation protocols, endogenous or spike-in controls for normalization of lncRNAs in saliva [162,163,164,165], uniform and consistent extraction methods, and differential centrifugation to fractionate saliva samples [166], as well as the increased credibility of qPCR results, with specificity and reliability (our unpublished data), to achieve more specific detection. Moreover, the development of fast, high throughput detection devices, such as biosensor chip technologies, would be critical for salivary lncRNA-based diagnosis and monitoring, and could greatly improve health care outcomes for oral cancer patients. At present, only limited circulating lncRNA biomarkers are approved for informing clinical decision making in other types of tumors. Moreover, for the identification of novel lncRNA biomarkers for oral cancer, sufficient clinical cohorts with matched clinical information are urgently needed to validate the performance of these RNA-based biomarkers for early-diagnosis, prognosis, and drug response. 

Nevertheless, in the last decade, accumulating GWASs (Genome-wide association study) studies have identified more than 6500 disease-predisposing SNPs, and only 7% are located in protein-coding regions. Remarkably, structural approaches have revealed that a few SNPs and somatic mutations within lncRNAs could disrupt local RNA structure at functionally relevant sites, affecting their molecular function and expression pattern, revealing that SNPs in cancer-associated lncRNAs may help to categorize patient populations [167]. Indeed, two SNPs within lncRNA H19, rs2839701 and rs217727, were identified related to OSCC susceptibility in a Chinese population [168]. However, to our knowledge, no direct evidence showing that the mutations/SNPs analyzed in OSCC could affect lncRNA secondary and tertiary structures was reported. Currently, the experimental approaches used to decipher RNA structure have been limited to small transcripts or RNA fragments, and unfeasible to scale to genome-wide analyses. Hence, the clinical translation and structural investigation of oral cancer-associated SNPs in GWAS data may warrant future research attention. 

Most excitingly, the aforementioned lncRNAs with key roles in oral tumorigenesis could be potential therapeutic targets. Knockdown of multiple specific oncogenic oral cancer-associated lncRNAs has been demonstrated to suppress various aspects of tumor progression and achieve a positive effect (Table 1.), revealing that targeting these transcript-involved axes might be new therapeutic option. In light of their sensitivity, tissue-specificity, ease of design, and regulation of specific facets of cellular networks, lncRNAs may be superior to protein with regard to the undesired harmful adverse effects associated with their targeting. Moreover, owing to the lack of translation into proteins, rapid turnover rate, and generally lower expression levels of lncRNAs, targeting lncRNAs suggests fast effects with low doses.

To date, the most advanced and straightforward therapeutic attempts at RNA targeting have been accomplished through direct targeting of sequences via antisense oligonucleotides (ASOs) and siRNA-based therapeutics. Basically, according to sequence homology and RNA accessibility, these oligonucleotide-based molecules can be quickly designed and bind to the target RNA through Watson–Crick base pairing to induce co-transcriptional cleavage or translation repression [169,170]. In the past few years, both antisense oligonucleotides (ASOs) and siRNA have received FDA approval for first-of-its-kind mRNA-targeting in Mipomersen (Kynamro™) and patisiran (ONPATTRO™) for two nonmalignant diseases, familial hypercholesterolemia [171] and transthyretin amyloidosis [172], respectively. Since then, several mRNA-targeting ASOs and siRNAs have been approved, revealing the promise of RNA therapeutics; though neither have yet been proven as an anticancer therapy. Due to in vivo adverse effects and the lack of appropriate delivery systems, there are still limitations to using ASOs and siRNAs in the clinic. A series of chemical modifications and variations have been introduced to improve their pharmacological properties, such as enhancing hybridization affinity to the target RNA, increasing resistance to nuclease-mediated degradation and reducing non-specific immunostimulatory activity [169,170]. However, most of the delivery systems and targeting strategies have been applied to mRNAs. On the contrary, the experience with therapeutic lncRNA-targeting is extremely limited. Currently, several ASO- and siRNA-targeting oncogenic lncRNAs are under development and protected by patents. There are both advantages and challenges ahead for lncRNA-targeting therapy. With a deep understanding of lncRNA localization, structures, functional motifs, mechanisms of action, and interplay with other biological molecules, it is believed that lncRNA-based therapy will surely bring disruptive innovation to the field of cancer therapy.

## 5. Conclusions and Perspectives

Over the past decade, with the advent of various genomic technologies, profiling investigations of lncRNAs in clinical tissue samples have revealed the key role played by lncRNAs in oral tumorigenesis; filling current research gaps in our understanding of cancer biology. This review has presented an updated summary of our current knowledge regarding dysregulated lncRNA profiles and their mechanisms of action in oral cancer (Table 1 and Figure 1), highlighting the functional annotation and regulatory role of these oral cancer-associated lncRNAs in profound oncogenic signaling pathways (Figure 2). Collectively, these recent discoveries in oral cancer have strengthened the idea that long non-coding RNA indeed acts as the molecular functional code to fine tune cellular gene expression. LncRNAs can participate in the development and progression of oral cancer at diverse levels, including epigenetic, transcriptional, and post-transcriptional steps via an elaborate network consisting of various kinds of RNA, chromatin DNA, and protein factors, although the underlying mechanisms of most oral cancer-associated lncRNAs remain largely unknown. 

In recent years, for healthcare research, the development of non-invasive approaches to monitoring health states, tumor progression, and post-treatment response has become one of the most desirable goals. The current studies of lncRNA in oral cancer may facilitate the identification of valuable and convenient biomarkers to improve the diagnosis of high-risk patients with premalignant lesions or previous history of cancer. Encouragingly, recent investigations combined with statistics have identified multiple circulating lncRNAs detectable in blood and saliva harboring the potential to serve as non-invasive diagnostic and prognostic biomarkers for oral cancer. It is expected that more will be discovered in the near future, however, further extensive research, along with prospective studies with large cohorts, will be needed to validate these results and the performance of these biomarkers in oral cancer, to make liquid biopsy tests reliably translate into the clinic routine in oral cancer diagnostics.

In summary, our current knowledge has affirmed the pivotal role of lncRNAs in basic, translational, and clinical oral oncology. To fully explore the molecular mechanism and clinical applications of lncRNAs in oral cancer requires an advancement in analytical methodologies for deeper investigation and comprehension. Moreover, the integration of lncRNA profiling data in oral cancer using bioinformatics would enhance our understanding of regulatory signaling pathways in this malignancy. In the context of personalized medicine, it is anticipated that oral cancer-related lncRNAs will gain greater relevance and recognition, and we hope to see the rapid expansion of lncRNA-based clinical tools in the next decade, to significantly benefit oral cancer patients.

## Figures and Tables

**Table 1:** LncRNAs in oral cancer.

lncRNA	Expression Levels in Cancer	Clinical Association	Functional Regulation	Interactor	Target/Effect	Mechanistic Classification	Refs
AC007271.3*	Upregulated	Expressed in serum and tissues of OSCC patients Associated with clinical stage, lymph node metastasis (LNM), poor histological differentiation, and unfavorable prognosis	↑ Proliferation ↑ Migration ↑ Invasion ↓ Apoptosis	N.D.	β-catenin and its downstream target molecules CyclinD1, c-myc and Bcl-2	**Unclear mechanism**	[69,70]
ANRIL*	Upregulated	Expressed in serum and tissues of OSCC patients Overexpressed during carcinogenesis and correlated with both high TNM stage and lymph node metastasis (LNM)	↑ Proliferation ↑ Migration ↑ Invasion ↓ Apoptosis ↓ Cisplatin cytotoxicity	miR-125a	Estrogen Related Receptor α (ESRRA)	**Sequestration of** **miRNAs** (*ANRIL* acts as a miR-125a sponge, thereby enhancing ESRRA expression)	[56]
				N.D.	TGF-β/Smad signaling pathway	**Unclear mechanism**	[71]
				N.D.	Drug transporters (MRP1 and ABCC2)	**Unclear mechanism**	[72]
***BC200***	Upregulated	N.D.	↑ Proliferation ↑ Migration	N.D.	MMP-9 and MMP-13 expression	**Unclear mechanism**	[73]
***BLACAT1***	Upregulated	N.D.	↑ Viability ↑ Migration ↑ Invasion	miR-142-5p	N.D.	**Sequestration of** **miRNAs** (*BLACAT1* acts as a miR-142-5p sponge)	[74]
***C5orf66-AS1***	Downregulated	N.D.	↓ Cell growth ↓ Metastasis	N.D.	CYC1 expression	**Unclear mechanism**	[24]
***CASC2***	Downregulated	Correlated with tumor size and adverse clinicopathological characteristics of OSCC patients Expression was increased in patients without recurrence	↓ Migration ↓ Invasion ↓ Proliferation ↑ Apoptosis	N.D.	Downregulation of CDK1	**Unclear mechanism**	[25]
				miRNA-21	PDCD4 expression	**Sequestration of miRNAs** (***CASC2*** acts as a miRNA-21 sponge, thereby enhancing PDCD4 expression)	[27]
CASC9*	Upregulated	Associated with tumor size, clinical stage, regional lymph node metastasis, and overall survival time in OSCC patients	↑ Proliferation ↓ Autophagy-mediated cell apoptosis	N.D.	AKT/mTOR pathway	**Unclear mechanism**	[75]
***CASC15***	Upregulated	Expressed in plasma of stage I and II OSCC patients Inversely correlated with lncRNA MEG3 in OSCC tissues	↑ Proliferation	N.D.	MEG3	**Unclear mechanism**	[76]
CCAT1*	Upregulated	N.D.	↑ Proliferation ↑ Migration ↑ Invasion ↓ Apoptosis	miR-181a	Wnt/β-catenin signaling	**Sequestration of miRNAs** (*CCAT1* acts as a miRNA sponge)	[77,78]
				miR155-5p and let7b-5p	N.D.		
***CCHE1***	Upregulated	N.D.	↑ Proliferation ↑ Migration ↑ Invasion ↓ Apoptosis	miR-922	PAK2 expression	**Sequestration of miRNAs** (***CCHE1*** acts as a miR-922sponge, thereby enhancing PAK2 expression)	[79]
CEBPA-AS1*	Upregulated	Correlated with poor differentiation, lymph node metastasis, and high clinical stage	↑ Proliferation ↑ Migration ↑ Invasion ↓ Apoptosis	CEBPA	Bcl-2 expression	**Post-transcriptional regulation** (*CEBPA-AS1* might form a “lncRNA-mRNA” pair with CEBPA and regulate CEBPA expression in a *cis* manner)	[48]
***CRNDE***	Upregulated	N.D.	↑ Proliferation ↑ Migration ↑ Invasion ↑ EMT ↓ Apoptosis	N.D.	Wnt/β-catenin signaling	**Unclear mechanism**	[80]
***DANCR***	Upregulated	Correlated with higher clinical stage, lower differentiation degree, or lymph node metastasis	↑ Proliferation ↑ Migration ↑ Invasion ↓ Apoptosis	miR-216a-5p	Bcl-2 expression KLF12 expression	**Sequestration of miRNAs** (*DANCR* acts as a miR-216a-5p sponge, thereby enhancing Bcl-2 and KLF12 expression)	[81]
***DNM3OS***	Upregulated	N.D.	↑ Viability ↑ Migration	miR-204-5p	HIP1 expression	**Sequestration of miRNAs** (*DNM3OS* acts as a miR-204-5p sponge, thereby enhancing HIP1 expression)	[82]
***ELF3-AS1***	Upregulated	N.D.	↑ Proliferation	N.D.	GLUT1 expression	**Unclear mechanism**	[83]
***ENST00000470447.1***	Downregulated	Correlated with tumor differentiation High expression was associated with better disease-free survival for patients	↓ Proliferation ↓ Migration ↓ Invasion ↑ Apoptosis	N.D.	N.D.	**Unclear mechanism**	[28]
***FAL1***	Upregulated	N.D.	↑ Proliferation	miR-761	CRKL expression	**Sequestration of miRNAs** (*FAL1* acts as a miR-761 sponge, thereby enhancing CRKL expression)	[84]
FALEC *	Downregulated	N.D.	↓ Proliferation ↓ Migration	EZH2	ECM1 expression	**Epigenetic and transcriptional regulation** (*FALEC* inhibited transcription through recruiting EZH2 to the promoter of ECM1)	[29]
FGD5-AS1 *	Upregulated	N.D.	↑ Proliferation ↑ Migration ↑ Invasion ↓ Apoptosis	miR-153-3p	MCL1 expression	**Sequestration of miRNAs** (*FGD5-AS1* acts as a miRNA sponge, thereby enhancing the expression of their targets)	[57,85]
				miR-520b	USP21 expression		
GAS5 *	Downregulated	N.D.	↓ Proliferation ↓ Migration ↓ Invasion ↓ EMT	miR-21	Regulation of PI3K/Akt pathwayPTEN expression	**Sequestration of miRNAs** (*GAS5* acts as a miR-21 sponge, thereby enhancing PTEN expression)	[86,87]
H19 *	Upregulated	Associated with the TNM stage, nodal invasion, and shorter overall survival of patients	↑ Proliferation ↑ Migration ↑ Invasion ↑ EMT ↓ Apoptosis	miR-138	EZH2 expression	**Sequestration of miRNAs** (*H19* acts as a miR-138 sponge, thereby enhancing EZH2 expression)	[58]
HAS2-AS1 *	Upregulated	Associated with lymph node metastasis and hypoxic tumor status in patients	↑ Invasion ↑ EMT	HAS2 gene	Transcription of HAS2	**Transcriptional regulation** (*HAS2-AS1* is necessary for the transcription of its sense counterpart *HAS2* upon hypoxia treatment)	[41]
***HCP5***	Upregulated	Associated with the aggressive clinicopathological characteristics and poor prognosis of patients	↑ Proliferation ↑ Migration ↑ Invasion ↑ EMT	miR-140-5p	SOX4 expression	**Sequestration of miRNAs** (*HCP5* acts as a miR-140-5p sponge, thereby enhancing SOX4 expression)	[88]
***HIFCAR/*** MIR31HG *	Upregulated	Associated with age and advanced tumor grade	↑ Tumor progression ↑ Metastatic potential	HIF-1α	HIF-1α signaling	**Transcriptional regulation** (*LncHIFCAR* acts as HIF-1α coactivator)	[43]
					p21	**Unclear mechanism**	[44]
HNF1A-AS1 *	Upregulated	High expression predicted poor prognosis for patients	↑ Migration ↑ Invasion ↑ EMT	N.D.	Notch1 and Hes1 expression	**Unclear mechanism**	[89]
HOTAIR *	Upregulated	Correlated with TNM stage, histological grade, and differentiation, as well as regional lymph node metastasis Overexpression indicated poor overall survival and disease-free survival	↑ Proliferation ↑ Migration ↑ Invasion ↑ EMT ↓ Apoptosis ↑ Autophagy	EZH2 and H3K27me3	Regulation of E-cadherin	**Epigenetic and transcriptional regulation** (*HOTAIR* regulated E-cadherin expression through partly associating with EZH2 and mediating H3K27me3 at the promoter of E-cadherin)	[32]
				miR-326	MTA2 expression	**Sequestration of miRNAs** (*HOTAIR* acts as a miR-326 sponge, thereby enhancing MTA2 expression)	[37]
				N.D.	Regulation of mTOR and the autophagy-related factors	**Unclear mechanism**	[36]
***HOTTIP***	Upregulated	Associated with lymph node metastasis and late-stage OTSCC patients Correlated with poor prognosis	↑ Proliferation ↑ Migration ↑ Invasion ↑ EMT ↓ Apoptosis	miR-124-3p	HMGA2 expressionWnt/β-catenin pathway	**Sequestration of miRNAs** (*HOTTIP* acts as a miR-124-3p sponge, thereby enhancing HMGA2 expression)	[90]
				N.D.	Cell cycle arrest at G1 phase	**Unclear mechanism**	[91]
***HOXA11-AS***	Upregulated	N.D.	↑ Proliferation ↑ Migration ↑ Invasion ↑ EMT ↓ Apoptosis ↓ CDDP cytotoxicity	miR-98-5p	YBX2 expression	**Sequestration of miRNAs** (*HOXA11-AS* acts as a miRNA sponge, thereby enhancing the expression of their targets)	[92,93,94]
				miR-518a-3p	PDK1 expression		
				miR-214-3p	PIM1 expression		
***HOXC13-AS***	Upregulated	N.D.	↑ Proliferation ↑ Migration ↑ EMT	miR-378g	HOXC13 expression	**Sequestration of miRNAs** (*HOXC13-AS* acts as a miR-378g sponge, thereby enhancing HOXC13 expression)	[95]
***HULC***	Upregulated	N.D.	↑ Proliferation ↑ Migration ↑ Invasion ↓ Apoptosis ↑ EMT ↑ CDDP tolerance	N.D.	N.D.	**Unclear mechanism**	[96]
***JPX***	Upregulated	N.D.	↑ Proliferation ↑ Migration ↑ Invasion	miR-944	CDH2 expression	**Sequestration of miRNAs** (*JPX* acts as a miR-944sponge, thereby enhancing CDH2 expression)	[97]
LEF1-AS1*	Upregulated	Relevant to poor prognosis in OSCC	↑ Survival and proliferation ↑ Migration ↑ Invasion ↓ Apoptosis	LATS1	Regulation of Hippo signaling	**Interaction Decoy** (*LEF1-AS1* can interact with LATS1 and therefore regulates Hippo signaling)	[98]
***LINC00152***	Upregulated	Associated with decreased survival in patients	↑ Proliferation ↑ Colony formation ↑ Migration ↑ Invasion ↑ EMT	miR-139-5p	N.C.	**Sequestration of miRNAs** (*LINC00152* acts as a miR-139-5p sponge)	[99]
***LINC00319***	Upregulated	N.D.	↑ Proliferation ↑ Metastasis ↑ EMT ↑ Angiogenesis	miR-199a-5p	FZD4 expression	**Sequestration of miRNAs** (*LINC00319* acts as a miR-199a-5p sponge, thereby enhancing FZD4 expression)	[100]
LINC00941 *	Upregulated	N.D.	↑ Proliferation ↑ Colony formation	CAPRIN2	CAPRIN2 expression Canonical WNT/β-catenin signaling	**Transcriptional regulation** (*LINC00941* acts as transcriptional activator by looping to CAPRIN2 promoter)	[39]
***LINC00958***	Upregulated	Associated with poor prognosis	↑ Proliferation ↑ Invasion ↓ Apoptosis	miR-185-5p	YWHAZ expression	**Sequestration of miRNAs** (*LINC00958* acts as a miR-185-5p sponge, thereby enhancing YWHAZ expression)	[101]
***LINC00963***	Upregulated	N.D.	↑ CSC hallmarks ↑ Chemoresistance	N.D.	Stemness marker ALDH1Multidrug-resistance transporter ABCB5	**Unclear mechanism**	[102]
LINC01315 *	Downregulated	N.D.	↓ Proliferation ↓ Migration ↓ Invasion ↑ Apoptosis	miR-211	DLG3 expression Regulation of Hippo signaling pathway	**Sequestration of miRNAs** (*LINC01315* acts as a miR-211 sponge, thereby enhancing DLG3 expression)	[30]
LncRNA-p23154 *	Upregulated	N.D.	↑ Metastasis ↑ Glycolysis	Promoter region of miR-378a-3p	GLUT1 expression	**Transcriptional regulation** (*LncRNA-p23154* binds to the promoter region of miR-378a-3p)	[40]
***LUCAT1***	Upregulated	Associated with tumor size	↑ Cell growth ↑ Proliferation	N.D.	MAPK signaling	**Unclear mechanism**	[103]
MALAT1 *	Upregulated	Promotes OSCC progression	↑ Proliferation ↑ Migration ↑ Invasion ↑ EMT ↓ Apoptosis ↓ DDP-resistance	miR-143-3p	MAGEA9 expression	**Sequestration of miRNAs** (*MALAT1* acts as a miRNA sponge, thereby enhancing the expression of their targets)	[59,104,105,106]
				miR-140-5p	PAK1 expression		
				miR-125b	STAT3 expression		
				miR-101	EZH2 expression		
				N.D.	P-glycoprotein expression PI3K/AKT/m-TOR signaling	**Unclear mechanism**	[107,108]
				N.D.	Wnt/β-catenin signaling		
***MCM3AP-AS1***	Upregulated	Associated with poor prognosis in OSCC patients	↑ Proliferation ↑ Migration ↑ Invasion	miR-204-5p	FOXC1 expression	**Sequestration of miRNAs** (*MCM3AP-AS1* acts as a miRNA sponge, thereby enhancing the expression of their targets)	[109,110]
				miR-363-5p	N.D.		
MEG3 *	Downregulated	N.D.	↓ Proliferation ↓ Migration ↓ Invasion ↑ Apoptosis	miR-548d-3p	SOCS5 and SOCS6 expression Regulation of JAK-STAT signaling	**Sequestration of miRNAs** (*MEG3* acts as a miRNA sponge, thereby enhancing the expression of their targets)	[55,111,112]
				miR-21	N.D.		
				miR-361-5p	Regulation of succinate dehydrogenase (SDH)		
				N.D.	Wnt/β-catenin signaling	**Unclear mechanism**	[113]
***MIR4435-2HG***	Upregulated	N.D.	↑ EMT	miR-296-5p	Expression of EMT markers	**Sequestration of miRNAs** (*MIR4435-2HG* acts as a miR-296-5p sponge)	[114]
***MORT***	Downregulated	Low expression level was correlated with poor survival	↓ Proliferation	N.D.	ROCK1 expression	**Unclear mechanism**	[31]
***MYOSLID***	Upregulated	Advanced OSCC patients had higher MYOSLID expression levels than those in early stage patients	↑ Migration ↑ Invasion	N.D.	Expression of EMT-related markers	**Unclear mechanism**	[115]
***NEAT1***	Upregulated	Correlated with aggressive tumor phenotypes and poor prognosis	↑ Proliferation ↑ Migration ↑ Invasion ↓ Apoptosis	miR-365	RGS20 expression	**Sequestration of miRNAs** (*NEAT1* acts as a miR-365 sponge, thereby enhancing RGS20 expression)	[116]
***OIP5-AS1***	Upregulated	Overexpression in oral tumors with undifferentiated cellular pathology Overexpression is common in human cancers of epithelial origin	↑ Proliferation ↑ Migration↑ Invasion	miR-338-3p	NRP1 expression	**Sequestration of miRNAs** (*OIP5-AS1* acts as a miRNA sponge, thereby enhancing the expression of their targets)	[117,118]
				miR-137 miR148a-3p miR-30a-5p miR-30b-5p miR-338-3p miR-22-3p	N.D.		
PAPAS *	Upregulated	Associated with worse survival conditions	↑ Migration ↑ Invasion	N.D.	TGF-β signaling	**Unclear mechanism**	[119]
PLAC2 *	Upregulated	N.D.	↑ Proliferation ↑ Invasion	N.D.	Wnt/β-catenin signaling	**Unclear mechanism**	[120]
PDIA3P *	Upregulated	Related to poorer prognosis	↑ Proliferation	miR-185-5p	CCND2 expression	**Sequestration of miRNAs** (*PDIA3P* acts as a miR-185-5p sponge, thereby enhancing CCND2 expression)	[60]
***PVT1***	Upregulated	Correlated with worse overall survival Frequently upregulated in cisplatin-resistant tissues	↑ Proliferation ↑ Migration ↑ Invasion ↑ Cisplatin-resistance ↓ Apoptosis	miR-150-5p	GLUT-1 expression	**Sequestration of miRNAs** (*PVT1* acts as a miRNA sponge, thereby enhancing the expression of their targets)	[121,122]
				miR-194-5p	HIF-1α expression		
RBM5-AS1 *	Upregulated	N.D.	↑ Proliferation ↑ Migration ↑ Invasion	miR-1285-3p	YAP1 expression	**Sequestration of miRNAs** (*RBM5-AS1* acts as a miR-1285-3p sponge, thereby enhancing YAP1 expression)	[61]
RC3H2 *	Upregulated	N.D.	↑ Cell growth ↑ Colony formation ↑ Migration ↑ Invasion	miR-101-3p	EZH2 expression	**Sequestration of miRNAs** (*RC3H2* acts as a miR-101-3p sponge, thereby enhancing EZH2 expression)	[53]
***RP11-874J12.4***	Upregulated	N.D.	↑ Proliferation ↑ Migration	miR-19a-5p	EBF1 expression	**Sequestration of miRNAs** (*RP11-874J12.4* acts as a miR-19a-5p sponge, thereby enhancing EBF1 expression)	[123]
***SNHG12***	Upregulated	N.D.	↑ Proliferation ↑ Migration ↑ Invasion ↓ Apoptosis	miR-326	E2F1 expression	**Sequestration of miRNAs** (*SNHG12* acts as a miR-326 sponge, thereby enhancing E2F1 expression)	[124]
***SNHG17***	Upregulated	Correlated with unfavorable pathological indexes	↑ Proliferation ↑ Migration ↑ Invasion ↓ Apoptosis	miR-375	PAX6 expression	**Sequestration of miRNAs** (*SNHG17* acts as a miR-375 sponge, thereby enhancing PAX6 expression)	[125]
SNHG20 *	Upregulated	Associated with tumor differentiation and TNM stage Higher expression predicted a poor overall survival (OS) rate in patients	↑ Proliferation ↑ Migration ↑ Invasion ↑ CSC properties	miR-29a	DIXDC1 expression Regulation of Wnt signaling	**Sequestration of miRNAs** (*SNHG20* acts as a miRNA sponge, thereby enhancing the expression of their targets)	[54,126,127]
				miR-197	LIN28 expression		
SNHG3 *	Upregulated	N.D.	↑ Proliferation ↑ Migration	ELAVL1	NFYC expression Wnt/β-catenin signaling	**Protein scaffold** (*SNHG3* can bind to ELAVL1 and therefore stabilize and upregulate NFYC expression)	[66]
				miR-2682-5p	HOXB8 expression	**Sequestration of miRNAs** (*SNHG3* acts as a miR-2682-5p sponge, thereby enhancing HOXB8 expression)	[128]
***SOX21-AS1***	Downregulated	Correlated with an advanced stage, large tumor size, and poor disease-specific survival	↓ Cell growth ↓ Invasion	N.D.	N.D.	**Unclear mechanism**	[129]
TIRY *	Upregulated	Higher expression levels in OSCC tissues predicted poor prognosis	↑ Proliferation ↑ Migration ↑ Invasion ↑ EMT	miR-14	Expression of EMT markers Wnt/β-catenin signaling	**Unclear mechanism**	[130]
TTN-AS1 *	Upregulated		↑ Proliferation ↑ Migration ↓ Apoptosis	miR-411-3p	NFAT5 expression	**Sequestration of miRNAs** (*TTN-AS1* acts as a miR-411-3p sponge, thereby enhancing NFAT5 expression)	[63]
TUG1 *	Upregulated	Correlated with TNM stage, lymph node metastasis, and tumor grade in patients	↑ Proliferation ↑ Migration ↑ Invasion	miR-524-5p	DLX1 expression	**Sequestration of miRNAs** (*TUG1* acts as a miR-524-5p sponge, thereby enhancing DLX1 expression)	[62]
				N.D.	Wnt/β-catenin signaling	**Unclear mechanism**	[131]
UCA1 *	Upregulated	Associated with lymph node metastasis and TNM stage	↑ Proliferation ↑ Migration ↑ Invasion ↑ Chemoresistance↓ Apoptosis	miR-143-3p	MYO6 expression	**Sequestration of miRNAs** (*UCA1* acts as a miRNA sponge, thereby enhancing the expression of their targets)	[132,133]
				miR-184	SF1 expression		
				N.D.	Regulation of cisplatin-activated PI3K/Akt signaling	**Unclear mechanism**	[134,135]
				N.D.	Wnt/β-catenin signaling		

* The lncRNAs discussed in the context of this review.

## Abbreviations

ASO	Antisense oligonucleotide
Bcl-2	B-cell lymphoma 2
CAPRIN2	Caprin family member 2
ceRNA	Competing endogenous RNA
CTCF	CCCTC-binding factor
EMT	Epithelial-mesenchymal transition
EZH2	Enhancer of zeste homolog 2
GLUT1	Glucose transport 1
H3K27me3	Histone H3 lysine 27 trimethylation
Hes1	Hairy and enhancer of split 1
HIF-1α	Hypoxia-inducible factor 1-alpha
HNSCC	Head and neck squamous cell carcinoma
JAK	Janus kinases
LC3B	Microtubule-associated protein 1-light chain 3 beta
LATS1	Large tumor suppressor kinase 1
LncRNA	Long non-coding RNA
miRNA	microRNA
MOB	Monopolar spindle-one-binder protein
Notch1	Neurogenic locus notch homolog protein
OSCC	Oral squamous cell carcinoma
PRC2	Polycomb repressive complex 2
RBP	RNA binding protein
SOCS	Suppressor of cytokine signaling
STAT	Signal transducer and activator of transcription
TGF-β	Transforming growth factor-β
TSCC	Tongue squamous cell carcinoma
UTR	Untranslated region
YAP1	Yes-associated protein 1
ELAVL1	Embryonic lethal abnormal vision-like protein 1
HuR	Hu antigen R
NFYC	Nuclear transcription factor Y subunit gamma
APC	Adenomatous polyposis coli
CK1	Casein kinase 1
Dvl	Dishevelled
Fzd	Frizzled
GSK-3β	Glycogen synthase kinase-3β
MMP-9	Matrix metallopeptidase 9
LEF	Lymphoid-enhancing factor
LRP	Lipoprotein receptor-related protein
TCF	T-cell factor
AKT	Protein kinase B
mTOR	Mammalian target of rapamycin
PI3K	Phosphoinositide 3-kinase
PIP_2_	Phosphatidylinositol 4,5-bisphosphate
PIP_3_	Phosphatidylinositol 3,4,5-triphosphate
PTEN	Phosphatase and tensin homolog
RTK	Receptor tyrosine kinase
